# QuickStats

**Published:** 2013-07-19

**Authors:** Jiaquan Xu

**Figure:**
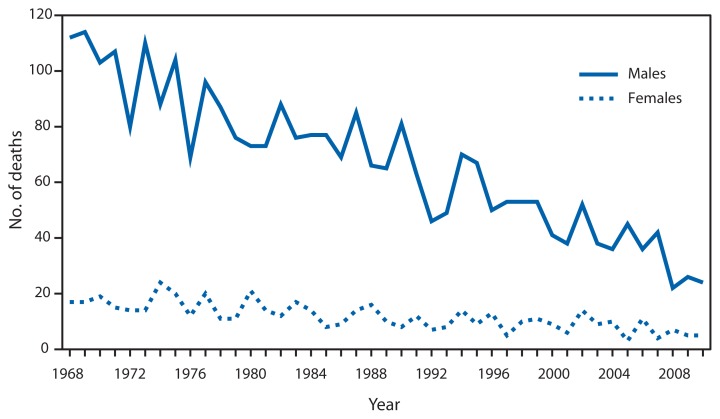
Number of Deaths^*^ from Lightning Among Males and Females — National Vital Statistics System, United States,^†^ 1968–2010 ^*^ Deaths from lightning (excluding deaths from fire caused by lightning, or injury from fall of tree or fall of other object caused by lightning), with lightning as the underlying cause of death, are coded as X33 (1999–2010) according to the *International Classification of Diseases, 10th Revision* and E907 (1968–1998) according to the *International Classification of Diseases, Ninth and Eighth Revisions*. ^†^Among U.S. residents only.

From 1968 to 2010, deaths from lightning in the United States decreased by 78.6% among males and 70.6% among females. During this 43-year period, a total of 3,389 deaths from lightning were recorded, an average of 79 per year. The highest yearly total of deaths from lightning (131) was recorded in 1969, and the lowest total (29) was recorded in 2008 and again in 2010. During the period, 85.0% of lightning deaths were among males.

**Source:** National Vital Statistics System. Mortality public use data files, 1968–2010. Available at http://www.cdc.gov/nchs/data_access/vitalstatsonline.htm.

